# miRNA signature of unfolded protein response in H9c2 rat cardiomyoblasts

**DOI:** 10.1186/2045-3701-4-56

**Published:** 2014-09-19

**Authors:** Danielle E Read, Ananya Gupta, Yury Ladilov, Afshin Samali, Sanjeev Gupta

**Affiliations:** Discipline of Pathology, School of medicine, Clinical Science Institute, National University of Ireland Galway, Galway, Ireland; Center for Cardiovascular Research, Charité-Universitätsmedizin Berlin, Berlin, Germany; Apoptosis Research Centre, School of Natural Sciences, National University of Ireland Galway, Galway, Ireland

**Keywords:** Unfolded protein response, ER stress, MicroRNAs, Myocardial infarction, Mir-7a, Apoptosis

## Abstract

**Background:**

Glucose and oxygen deprivation during ischemia is known to affect the homeostasis of the endoplasmic reticulum (ER) in ways predicted to activate the unfolded protein response (UPR). Activation of UPR signalling due to ER stress is associated with the development of myocardial infarction (MI). MicroRNAs (miRNAs) are key regulators of cardiovascular development and deregulation of miRNA expression is involved in the onset of many cardiovascular diseases. However, little is known about the mechanisms regulating the miRNA expression in the cardiovascular system during disease development and progression. Here we performed genome-wide miRNA expression profiling in rat cardiomyoblasts to identify the miRNAs deregulated during UPR, a crucial component of ischemia.

**Results:**

We found that expression of 86 microRNAs changed significantly during conditions of UPR in H9c2 cardiomyoblasts. We found that miRNAs with known function in cardiomyoblasts biology (miR-206, miR-24, miR-125b, miR-133b) were significantly deregulated during the conditions of UPR in H9c2 cells. The expression of miR-7a was upregulated by UPR and simulated *in vitro* ischemia in cardiomyoblasts. Further, ectopic expression of miR-7a provides resistance against UPR-mediated apoptosis in cardiomyoblasts. The ample overlap of miRNA expression signature between our analysis and different models of cardiac dysfunction further confirms the role of UPR in cardiovascular diseases.

**Conclusions:**

This study demonstrates the role of UPR in deregulating the expression of miRNAs in MI. Our results provide novel insights about the molecular mechanisms of deregulated miRNA expression during the heart disease pathogenesis.

## Background

Physiological or pathological processes that disturb protein folding in the endoplasmic reticulum cause ER stress and activate a set of signalling pathways termed the Unfolded Protein Response (UPR). In the ischemic state the lack of oxygen and nutrients to the heart can cause lasting damage to this vital organ through cardiomyoblasts death [1]. Ischemic conditions are known to affect ER homeostasis in ways predicted to activate the UPR [2]. Activation of UPR signalling due to ER stress is associated with the development of ischemic heart disease [3]. We and others have shown that simulating ischemia or ischemia/reperfusion in cultured neonatal rat or adult mouse ventricular cardiomyocytes can activate numerous features of the UPR [4].

In mammals, three ER transmembrane proteins, IRE1, ATF6, and PERK, respond to the accumulation of unfolded proteins in the ER lumen [5]. Activation of PERK, IRE1, and ATF6 initiates ER-to-nucleus intracellular signaling cascades collectively termed the UPR. PERK-mediated phosphorylation of eukaryotic translation initiation factor 2 on the alpha subunit (eIF2α) at Ser51 leads to translational attenuation [6]. Whilst phosphorylation of eIF2α inhibits general translation initiation, it paradoxically increases translation of activating transcription factor 4 (ATF4), which induces the transcription of genes involved in restoration of ER homeostasis [7]. The endoribonuclease activity of IRE1 is responsible for the nonconventional splicing of transcription factor XBP1, which controls the transcription of chaperones and genes involved in ER-associated protein degradation (ERAD) [8]. In response to ER stress, ATF6 translocates to the Golgi complex and is sequentially cleaved by two proteases [9]. The processed form of ATF6 (the activated transcription factor) subsequently translocates to the nucleus and binds to ATF/cAMP response elements (CRE) and ER stress responsive elements (ERSE-1) to activate target genes. The transcription factor C/EBP homologous protein (CHOP) operates as a downstream component of ER stress pathways and can transcriptionally upregulate expression of BIM (pro-apoptotic member of the BCL-2 family) during conditions of ER stress [10]. Thus, the UPR attempts to restore ER homeostasis by increasing ER biogenesis, decreasing the influx of new proteins into the ER, promoting transport of damaged proteins from the ER to the cytosol for degradation, and upregulating protein folding chaperones [5]. However, if the damage is too severe and ER homeostasis cannot be restored, apoptosis ensues [11]. Recently we have shown that small 20–22-nt RNAs, commonly referred to as microRNAs (miRNAs), play an important role in the regulation of life and death decisions following ER stress [12].

miRNAs have been shown to be critically involved in control of cell survival and cell death decisions [13–15]. miRNAs are generated from RNA transcripts that are exported into the cytoplasm, where the precursor-miRNA molecules undergo Dicer-mediated processing (removal of the hairpin loop) to generate mature miRNA [16]. The mature miRNAs assemble into RNA-induced silencing complexes (RISCs) and guide the silencing complex to specific mRNA target molecules with the assistance of argonaute proteins. The main function of miRNAs is to direct posttranscriptional regulation of gene expression, typically by binding to the 3’ UTR of cognate mRNAs and inhibiting their translation and/or stability by targeting them for degradation [17]. Several studies have shown global alterations in miRNA-expression profiles during various types of cellular stresses, such as folate deficiency, arsenic exposure, hypoxia, drug treatment and genotoxic stress [18]. Argonaute family member Ago2, a vital component of RISCs, is distributed diffusely in the cytoplasm and redistributes from the cytoplasm to stress granules and processing (P)-bodies upon exposure to stress conditions [19]. Stress-induced enrichment of Ago2 from cytoplasm to P-bodies is dependent on mature miRNAs suggesting a link between miRNAs and cellular stress.

We performed genome-wide miRNA expression profiling in rat cardiomyoblasts during the conditions of UPR. We found that miRNAs (miR-206, miR-24, miR-125b, miR-133b) with known function in cardiomyoblasts biology [20–22] were significantly deregulated during the conditions of UPR in H9c2 cells. The expression of miR-7a was upregulated by UPR and simulated *in vitro* ischemia in cardiomyoblasts. Further, ectopic expression of miR-7a provides resistance against UPR-mediated apoptosis in cardiomyoblasts. This study demonstrates the role of UPR in deregulating the expression of miRNAs in MI. Our results provide novel insights about the molecular mechanisms of deregulated miRNA expression during the heart disease pathogenesis.

## Results and discussion

### Differential expression of miRNAs during UPR in H9c2 cells

**Figure 1:**
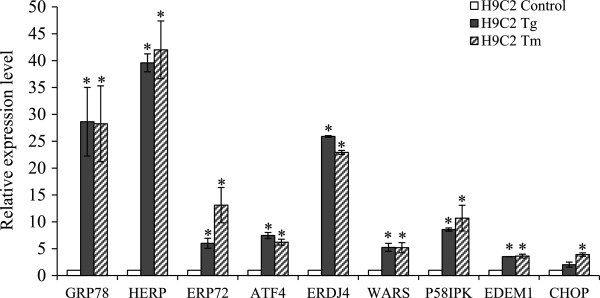
**Induction of UPR target genes in H9c2 cells.** H9c2 cells were left untreated or treated with (1 μM) Tg or (1 μg/ml) for 24 hours. The change in expression levels of ER stress markers was measured by qRT-PCR normalizing against GAPDH expression. The expression levels relative to the control are shown. Error bars represent mean ± S.D. from three independent experiments performed in triplicate. (*P < 0.05, two-tailed unpaired t-test compared with untreated cells).

MicroRNAs are important regulators of gene expression and we sought to identify miRNAs deregulated in the cellular response to UPR, a crucial component of ischemia. Treatment of H9c2 cells with the ER stressor thapsigargin (Tg), an inhibitor of the sacroplasmic/endoplasmic reticulum Ca^2+^-ATPase (SERCA) pump [23] and tunicamycin (Tm), an inhibitor of N-linked glycosylation [24] induced mRNA levels of many genes associated with the ER stress response (Figure 1). Next we profiled the expression of 350 mature rat miRNAs utilising a Sanger miRBase database (Release 11.0) μParaflo microfluidic chip (LC Sciences). This miRNA microarray platform generates reproducible data and is recommended for the study of changes in miRNA expression [25].

**Figure 2:**
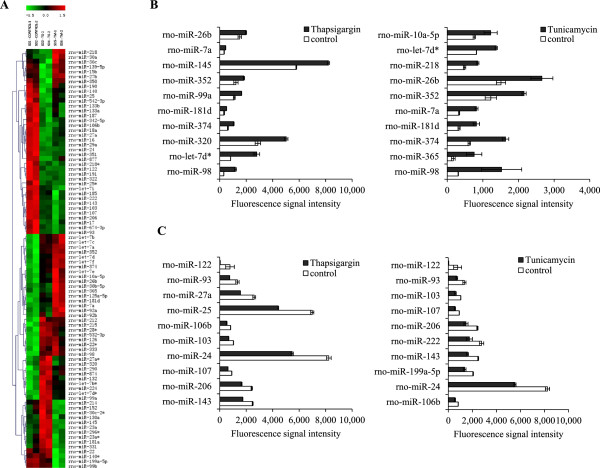
**miRNA expressions during unfolded protein response in H9c2 cardiomyocytes. (A)** Heat map summarizing the expression of miRNAs. Each column represents 1 of the samples, and each row represents 1 of 86 differentially expressed miRNAs at P <0.05. Samples were grouped by treatments, and miRNAs arranged by unsupervised hierarchical clustering. Red and green indicate up and downregulation, respectively, relative to the overall mean for each miRNA. Control-1 and control-2, untreated H9c2 cells; Tg-1 and Tg-2, thapsigargin treated H9c2 cells; Tm-1 and Tm-2, tunicamycin treated H9c2 cells. **(B)** Expression pattern of top 10 miRNAs (in terms of fluorescence intensity) upregulated upon treatment with thapsigargin and tunicamycin in H9c2 cells. **(C)** Expression pattern of top 10 miRNAs (in terms of fluorescence intensity) downregulated upon treatment with thapsigargin and tunicamycin in H9c2 cells.

Microarray analysis showed that out of 350 miRNAs spotted per chip, an average 198 miRNAs were detected. Further we found that expression of 86 (*p < 0.05*) miRNAs changed significantly during conditions of ER stress in H9c2 cardiomyoblasts (Figure 2A). We found that Tg treatment lead to a significant change in the expression of 48 (23 upregulated and 25 downregulated) miRNAs. Further Tm treatment lead to a significant change in the expression of 38 (16 upregulated and 22 downregulated) miRNAs. The top 10 miRNAs upregulated and downregulated upon treatment with Tg and Tm, respectively are shown in Figure 2B-C. The expression of miR-98, let-7d*, miR-374, miR-181d, miR-352, miR-7a and miR-26b were increased both by Tg and Tm in H9c2 cells. The expression of miR-122, miR-93, miR-103, miR-107, miR-206, miR-143, miR-24, and miR-106b were reduced upon treatment with Tg and Tm in H9c2 cells. There was 70% overlap among the top ten upregulated miRNAs by Tg and Tm and 80% overlap among the top ten downregulated miRNAs by Tg and Tm.

**Figure 3:**
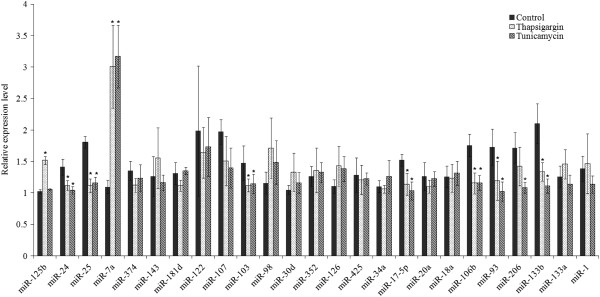
**Quantitative RT-PCR analyses of miRNAs deregulated during UPR in H9c2 cells.** H9c2 cardiomyocytes were either untreated (Control) or treated with (1.0 μM) Tg and (1.0 μg/ml) Tm for 24 hours. The expression level of indicated miRNAs was quantified by qRT-PCR, normalizing against snoRNA. Error bars represent mean ± S.D. from three independent experiments performed in triplicate. (* P < 0.05, two-tailed unpaired t-test compared with untreated cells).

To confirm the results of miRNA microarray analysis, we performed quantitative RT-PCR (qRT-PCR). Twenty five miRNAs were selected to span the range of high, medium, and low intensity signals. The four miRNAs (miR-125b, miR-30d, miR-34a and miR-1) were included as control miRNAs whose expression did not show significant change during conditions of UPR. The expression of these four miRNAs (miR-125b, miR-30d, miR-34a and miR-1) did not change during UPR, with exception of miR-125b whose expression was increased after Tg treatment (Figure 3). The effect of Tg on miR-125b expression is likely due to its effect on calcium homeostasis rather than UPR because Tm treatment had no effect on miR-125b expression. We observed that changes in the expression of nine miRNAs (miR-24, miR-25, miR-7a, miR-103, miR-17-5p, miR-106b, miR-93, miR-206 and miR-133b) analyzed by qRT-PCR were consistent with those by miRNA microarray at *p* < 0.05 (Figure 3). Further we found that expression of four miRNAs (miR-20a, miR-98, miR-107 and miR-126) showed a trend similar to that observed in microarray but was not statistically significant (Figure 3). We have recently shown that miRNAs belonging to the miR-106b-25 cluster are downregulated in a PERK-dependent manner and play an important role in ER stress-induced apoptosis [26]. In agreement with our previous results we observed reduced expression of all three miRNAs (mir-106b, miR25 and miR-93) belonging to the miR-106b-25 cluster during conditions of UPR in H9c2 cells. The miRNAs (miR-206, miR-24, miR-125b, miR-133b) deregulated upon UPR in H9c2 cells are abundantly expressed in adult heart. They belong to the top 20 most abundantly expressed miRNAs in murine adult heart as determined by number of normalised reads [27].

### Regulation of miR-7a expression by glucose deprivation and simulated ischemia

**Figure 4:**
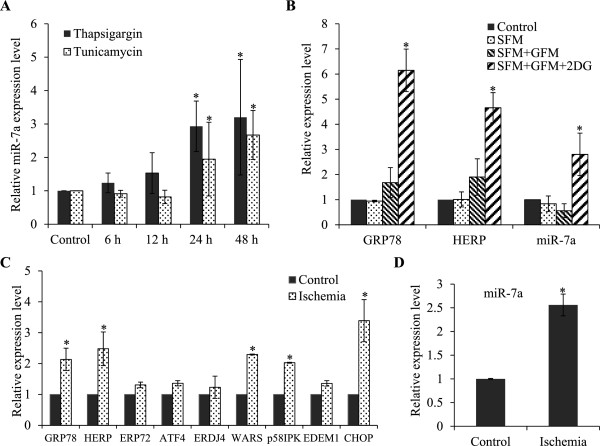
**Upregulation of miR-7a expression in primary cardiomyocytes during ischemia. (A)** H9c2 cardiomyocytes were either untreated (Control) or treated with (1.0 μM) Tg and (1.0 μg/ml) Tm for indicated time points. The expression level of indicated miRNAs was quantified by qRT-PCR, normalizing against snoRNA. Error bars represent mean ± S.D. from three independent experiments performed in triplicate. **(B)** H9c2 cells were treated with 2-deoxyglucose (1 mM) along with serum and glucose deprivation for 24 hours. The change in expression levels of ER stress markers normalized against GAPDH expression and miR-7a normalized against snoRNA expression was measured by qRT-PCR. The expression levels relative to the control are shown. SFM, serum free medium; GFM, glucose free medium and 2DG, 2-deoxyglucose. **(C-D)** Cardiomyocytes were exposed to simulated in vitro ischemia (Ischemia) consisting of glucose-free anoxia at pH 6.4 for 3 hours and cardiomyocytes not exposed to ischemia were used as controls (Control). The change in expression levels of ER stress markers normalizing against GAPDH expression **(C)** and miR-7a PCR normalizing against snoRNA expression **(D)** was measured by qRT-PCR. The expression levels relative to the control are shown. (*P < 0.05, two-tailed unpaired t-test compared with untreated cells).

We decided to focus on regulation of miR-7a by UPR in this study. Among nine miRNAs the deregulation of miR-7a was striking because of its significant increase upon Tg and Tm treatment when compared with untreated samples (Figure 3). We evaluated the change in miR-7a expression under various stress conditions by qRT-PCR. Notably, upon treatment with Tg and Tm the level of miR-7a increased in a time dependent manner (Figure 4A). Glucose deprivation is one of the crucial physiologic conditions leading to UPR activation, which is associated with several human diseases including tissue ischemia and cancer [4]. H9c2 cells were subjected to a combination of serum and glucose deprivation as described in materials and methods. We observed that glucose deprivation induced the expression of UPR target genes GRP78 and HERP (UPR target genes), thereby confirming the induction of UPR (Figure 4B) upon glucose deprivation. We found that conditions of glucose deprivation increased the levels of miR-7a in H9c2 cells (Figure 4B).

Next we determined the expression of miR-7a in primary culture of adult rat cardiomyoblasts during the conditions of *in vitro* simulated ischemia. In order to examine the effect of ischemia on the UPR, induction of UPR target genes was determined. Ischemia induced the expression of CHOP, WARS, p58^IPK^ and ERDJ4 (Figure 4C). Thapsigargin and Tunicamycin treatment also caused an increase in the expression of GRP78, HERP, CHOP, WARS and p58^IPK^ (Figure 1), although the level of mRNA induction was higher. Under similar conditions of *in vitro* simulated ischemia we observed a significant increase in the levels of miR-7a in primary cardiomyoblasts (Figure 4D). Collectively, these data confirmed that exposure of primary cardiomyoblasts to ischemic conditions induces UPR and miR-7a.

### miR-7a protects against UPR-induced cell death

**Figure 5:**
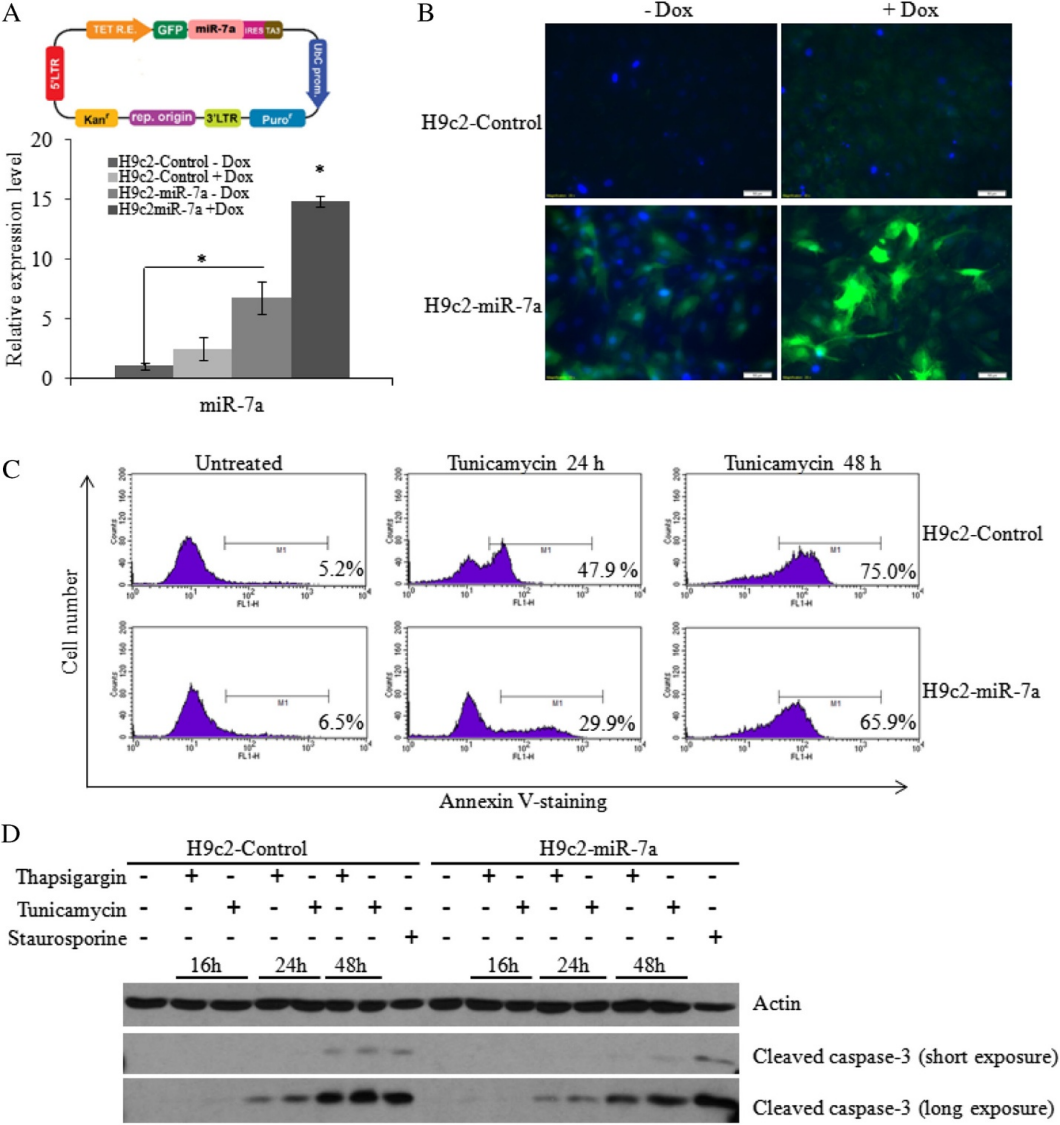
**Effect of miR-7a on UPR-mediated cell death. (A)** Upper panel, Shows a schematic representation of Lentiviral vector used to generate miR-7a expressing clones. Lower panel, H9c2-control and H9c2-miR-7a cells were treated with (1 μg/ml) of doxycycline for 24 h and expression levels of miR-7a was quantified by qRT-PCR, normalizing against snoRNA. Error bars represent mean ± S.D. from two independent experiments performed in triplicate. (*P < 0.05, two-tailed unpaired t-test as compared with uninduced cells). **(B)** Expression of GFP was monitored in H9c2-control and H9c2-miR-7a cells after treatment with (1 μg/ml) of doxycycline for 24 hours. **(C)** The H9c2-control and H9c2-miR-7a cells were treated (2 μg/ml) Tm for the indicated time. Histograms of annexinV-PE binding as obtained by FACS analysis of a representative experiment are shown. Numbers depict the percentage of annexin-positive cells. **(D)** The H9c2-control and H9c2-miR-7a cells were treated with (1 μM) Tg and (2 μg/ml) Tm for the indicated time and (1 μM) staurosporine for 24 hours. Western blotting of total protein was performed using antibodies against cleaved caspase-3 and actin.

Next we generated the clones of H9c2 cells expressing miR-7a to evaluate its role in ER stress-induced apoptosis. For this purpose H9c2 cells were transduced with tetracycline-inducible lentivirus engineered to produce GFP and miR-7a upon addition of tetracycline (Figure 5A) and co-expression of the tetracycline regulatory protein, TA3. Twenty-four hours after induction the H9c2-miR-7a clone exhibited significant expression of miR-7a, whereas no induction of miR-7a was observed in H9c2-control clones (Figure 5A). However we observed some transcriptional leakage even in the absence of doxycycline inducer in the H9c2-miR-7a clone, as determined by the expression of GFP and miR-7a in the absence of doxycycline (Figure 5A-B). Therefore H9c2-control and H9c2-miR-7a clones supplemented the doxycycline (1 μg/ml) were used in subsequent experiments. Western blotting for cleaved caspase-3 revealed that treatment with Tg and Tm induced apoptosis in both H9c2-control and H9c2-miR-7a cells. The extent of ER stress-induced apoptosis was decreased in H9c2-miR-7a cells as compared to H9c2-control cells (Figure 5C-D). However, there was no difference in the staurosporine-induced apoptosis between H9c2-control and H9c2-miR-7a cells (Figure 5D). Thus, overexpression of miR-7a appears to protect H9c2 cells against ER stress-induced apoptosis.

**Figure 6:**
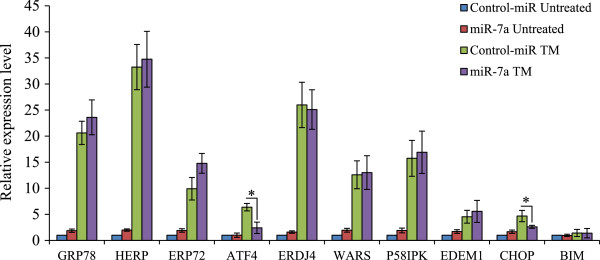
**Effect of miR-7a on induction of UPR-target genes.** H9c2 cells transfected with control miR or miR-7a were left untreated or treated with (1 μg/ml) for 24 hours. The change in expression levels of ER stress markers was measured by qRT-PCR normalizing against GAPDH expression. The expression levels relative to the untreated control are shown. (*P < 0.05, two-tailed unpaired t-test).

A variety of transcription factors activated during UPR collaborate to induce the expression of a wide array of targets that include ER chaperones and genes involved in ERAD to enhance the protein folding capacity of the cell and to decrease the unfolded protein load of the ER [5]. To investigate the basis for the reduced ER stress-induced cell death associated with expression of miR-7a, we compared the induction of key UPR target genes [28, 29] in control and pre-miR-7a transfected H9c2 cells. The qRT-PCR showed that induction of ATF4 and CHOP was significantly attenuated in pre-miR-7a transfected cells as compared to control transfected cells (Figure 6) upon treatment with tunicamycin. However, there was no difference in the induction of several other UPR-target genes such as GRP78, HERP, ERP72, ERDJ4, WARS p58IPK, EDEM1 and BIM (Figure 6). We did not find any binding site for miR-7a in the 3’ UTR of ATF4 or CHOP. Thus most likely the effects of miR-7a on induction of ATF4 and CHOP are indirect.

A growing body of evidence shows that miRNAs play an important role in heart diseases [30]. Several miRNAs have been implicated in the control of cardiac apoptosis and fibrosis following myocardial ischemia [31]. In this work, we report the extensive genome-wide profiling of miRNA expression in rat cardiomyoblasts during UPR, a crucial component of ischemia. We found that expression of many miRNAs (miR-24, miR-25, miR-7a, miR-103, miR-17-5p, miR-106b, miR-93, miR-206 and miR-133b) changed significantly during conditions of UPR in cardiomyoblasts. A similar alteration in expression level of these miRNAs has been previously reported by different research groups during conditions of idiopathic cardiomyopathy, ischemic cardiomyopathy, dilated cardiomyopathy, cardiac hypertrophy and heart failure [21, 30]. The muscle specific miR-1 and −206 are closely related in terms of expression and function. Both miR-1 and miR-206 are shown to promote myoblast-to-myotube differentiation [30, 32]. By contrast, miR-133 promotes the proliferation of myoblasts and inhibits their differentiation [20]. Further, miR-1 enhances cardiomyoblast apoptosis by targeting the expression of Hsp60 and Hsp70, while miR-133 targets and represses caspase-9 expression to decrease cardiomyoblast apoptosis [33]. The expression of miR-24 is down-regulated during MI and miR-24 regulates cardiomyoblast apoptosis, in part by direct repression of the BH3-only domain–containing protein Bim [22]. Further ectopic expression of miR-24 in a mouse MI model inhibited cardiomyoblast apoptosis, attenuated infarct size, and reduced cardiac dysfunction [22]. We have recently shown that miRNAs belonging to the miR-106b-25 cluster were downregulated during ER stress, in a PERK-dependent manner, and contributes to optimum induction of Bim and ER stress-induced cell death [26]. The ample overlap of microRNA expression signature between our analysis (in ER stress conditions) and different models of cardiac dysfunction further confirms the role of ER stress in cardiovascular diseases.

In the present study we investigated the potential role of miR-7a in ER stress-induced cell death. Previous studies have reported that miR-7a may act as tumour suppressor miRNA where it inhibits cell proliferation and increases cell apoptosis in some cancers [34]. miR-7a is expressed in a ventro-dorsal gradient along the ventricular wall and plays an important role in the determination of the dopaminergic phenotype during postnatal and adult olfactory neurogenesis by repressing Pax6 [35]. In addition miR-7a regulates pancreatic β-cell function by regulating the insulin granule exocytosis [36]. miR-7a is an IL-4-responsive gene in macrophages and functions to regulate IL-4-directed fusion of macrophages to form multinucleated giant cell [37]. However, the function of miR-7a in regulating cell fate during conditions of the UPR was not clear. We found that overexpression of miR-7a significantly decreased ER stress-induced cell apoptosis in cardiomyoblasts. The overexpression of miR-7a may protect rat cardiomyoblast against ER stress-induced cell apoptosis during MI. Indeed expression of miR-7a was shown to be upregulated in H9c2 cells after 10 h hypoxia and 2 h reoxygenation and transfection of miR-7a mimic significantly decreased cell apoptosis and cardiac infarct size in a rat I/R injury model [38]. However this is in contrast to previous reports where miR-7a has been shown to promote cancer progression by inhibiting cell proliferation and inducing apoptosis [30]. The miR-7a expression can modulate the activation of ATF4-CHOP signaling pathway during UPR. The overexpression of CHOP promotes apoptosis in several cell lines, whereas CHOP-deficient cells are resistant to ER stress-induced apoptosis [39]. Our results suggest that miR-7a expression abrogates induction of CHOP and thereby provide resistance to ER stress-induced cell death. However, we did not find any binding site for miR-7a in the 3’ UTR of ATF4 or CHOP. Thus most likely the effects of miR-7a on induction of ATF4 and CHOP are indirect. Our results warrant further studies to reveal the mechanism of ATF4-CHOP regulation by miR-7a.

## Conclusions

This study demonstrates the role of UPR in deregulating the expression of miRNAs in MI*.* The expression of miR-7a was upregulated by UPR and simulated *in vitro* ischemia in cardiomyoblasts. Further, ectopic expression of miR-7a provides resistance against UPR-mediated apoptosis in cardiomyoblasts. The ample overlap of miRNA expression signature between our analysis and different models of cardiac dysfunction further confirms the role of UPR in cardiovascular diseases.

## Methods

### Cell culture and treatments

The embryonic rat cardiac myoblasts H9c2 (ATCC, CRL-1446) was cultured in Dulbecco’s modified Eagle’s medium supplemented with 10% foetal bovine serum, 50 U/ml penicillin and 5 mg/ml streptomycin. To induce ER stress, cells were treated with 1 μM thapsigargin (Tg) or 1 μg/ml tunicamycin (Tm) for the indicated time periods. Glucose deprivation was achieved by changing the serum and glucose-containing DMEM to serum and glucose free-DMEM and (1 mM) 2-deoxyglucose for 24 hours.

### Generation of stable H9c2-miR-7a cells

We generated stable H9c2 cells with increased expression levels of miR-7a by using the lentiviral expression vector pLenti-III-Tet-mir (Applied Biological Materials Inc) and puromycin selection (3 μg/ml) for 7 days. This lentivector is designed to induce the expression of GFP and miRNA of interest upon addition of tetracycline.

### Nucleofection of H9c2 cells

The pre-miR precursor miRNAs (PM17111) Pre-miR control and (PM10047) Pre-miR-7a were purchased from Ambion. H9c2 cells were transfected with Pre-miR control and Pre-miR-7a by Nucleofection using nucleofactor kit L (Amaxa Nucleofector Technology) following the manufacturer’s instructions. 24 hours post transfection, the cells were treated with tunicamycin and total RNA was isolated at indicated time points.

### RNA extraction and real time RT-PCR

Total RNA was isolated using Trizol (Invitrogen) according to the manufacturer’s instructions. Reverse transcription (RT) was carried out with 2 μg RNA and Oligo dT (Invitrogen) using 20 U Superscript II Reverse Transcriptase (Invitrogen). For real-time PCR experiments, cDNA products were mixed with 2 × TaqMan master mix and 20 × TaqMan Gene Expression Assays (Applied Biosystems) and subjected to 40 cycles of PCR in StepOnePlus instrument (Applied Biosystems). Relative expression was evaluated with ΔΔCT method.

### miRNA microarray analysis

At 24 h post treatment with Tg or Tm, total RNA was isolated from the cell samples using the Trizol reagent according to the manufacturer’s instructions and quantified using a nanodrop at 260 nm. For both treatments, three independent biological replicates were generated. Briefly, the assay started with 5 μg of total RNA. Each total RNA sample was enriched for miRNAs. A 20 mer control RNA was spiked into each sample followed by labelling and hybridization. The control RNA was computationally and experimentally verified not to cross-hybridize with the probes of any known miRNA transcript. RNA samples were hybridized overnight on a μParaflo microfluidic chip. Each microfluidic chip contained 350 mature miRNAs of Rat as per Sanger miRBase database (Release 11.0). Each miRNA was spotted on the array nine times and for each RNA sample two chips were used. There were 16 sets of control probes on each array. There were >10 positive controls (spike-in controls & 5S). There were >10 negative controls (mismatch control). The background-subtracted signals were used for statistical tests and clustering analysis.

### Microarray data analysis

MiRNA microarray data were analyzed by LC Sciences by subtracting the background and normalizing the signals. Blank spaces represent signal values below detection level. A transcript to be listed as detectable must meet at least two conditions: signal intensity higher than 3 × (background standard deviation) and spot CV <0.5. CV is calculated by (standard deviation)/(signal intensity). When repeating probes are present on an array, a transcript is listed as detectable only if the signals from at least 50% of the repeating probes are above detection level. The miRNA microarray data used the total gene signal, which was the average value of repeating spots. During data process, “bad spots” that have signal values deviated more than 50% of average values of repeating spots and/or spot CV larger than 0.5 are discarded. Differentially expressed signals were determine by t-test with *p < 0.05*.

### Isolation of cardiomyoblasts and simulated ischemia

Ventricular cardiomyoblasts were isolated from male Wistar rats by perfusion of hearts with collagenase type II (300 U/mL) and cultured, as previously described [40]. For this purpose adult male rats were euthanized using deep isoflurane (5%) anaesthesia, hearts were rapidly excised, washed with ice-cold 0.9% NaCl and connected to the Langendorff-perfusion system. Anaesthesia depth was monitored by limb withdrawal using toe pinching. To separate cardiomyoblasts from non-cardiac cells, cardiomyoblasts were sedimented by low force and short centrifugations (5 g, 1 min, four times) and finally without centrifugation in medium containing 4% bovine serum albumin. To prevent growth of non-myocytes, medium was supplemented with 10 μmol/L cytosine-β-d-arabinofuranoside. After 1 hour of plating, cells were washed with culture medium (2% foetal calf serum) to remove non-attached cells. A high purity of cardiomyoblast culture (>93%) was confirmed by light microscopy. Third day after preparation cardiomyoblasts were exposed to simulated in vitro ischemia (SI) consisting of glucose-free anoxia at pH 6.4 as previously described [40]. After 3 hours of ischemia, total RNA was isolated and used for further analysis.

### TaqMan real-time microRNA PCR

Total RNA was reverse transcribed using the TaqMan miRNA Reverse Transcription Kit and miRNA-specific stem-loop primers (Applied BioSystems) in a small-scale RT reaction (comprised of 0.19 ml of H_2_O, 1.5 ml of 10X Reverse-Transcription Buffer, 0.15 ml of 100 mM deoxyribonucleotide triphosphates, 1.0 ml of Multiscribe Reverse-Transcriptase (50 U/ml), and 5.0 ml of input RNA (20 ng/ml); components other than the input RNA were prepared as a larger volume master mix), using a Tetrad2 Peltier Thermal Cycler (Bio-Rad, Alpha Technologies Ltd, Wicklow, Ireland) at 16°C for 30 min, 42°C for 30 min and 85°C for 5 min. For miRNAs and snoRNA, 4.0 ml of RT product was combined with 16.0 ml of PCR assay reagents (comprised of 5.0 ml of H_2_O, 10.0 ml of TaqMan 2X Universal PCR Master Mix, No AmpErase UNG, and 1.0 ml of TaqMan miRNA Assay) to generate a PCR of 20.0 μl of total volume. Real-time PCR was carried out on an Applied BioSystems 7900HT thermocycler at 95°C for 10 min, followed by 40 cycles of 95°C for 15 s and 60°C for 1 min. Data were analyzed with SDS Relative Quantification Software version 2.2.2 (Applied BioSystems.), with the automatic Ct setting for assigning baseline and threshold for Ct determination.

### Annexin V staining

Externalization of phosphatidylserine (PS) to the outer leaflet of the plasma membrane of apoptotic cells was assessed with annexin V-PE. Briefly, cells were collected by centrifugation at 350 g, washed once in ice-cold calcium buffer (10 mM HEPES/NaOH, pH 7.4, 140 mM NaCl, 2.5 mM CaCl2), and incubated with annexin V-FITC or with annexin V-PE for 15 minutes on ice. A wash step in calcium buffer was carried out prior to acquisition on a FACSCalibur flow cytometer (Becton Dickinson).

### Western blot

Cells were washed once in ice-cold PBS and lysed in whole cell lysis buffer (20 mM HEPES pH 7.5, 350 mM NaCl, 0.5 mM EDTA, 1 mM MgCl_2_, 0.1 mM EGTA and 1% NP-40) after stipulated time of treatments and boiled at 95°C with Laemmli’s SDS-PAGE sample buffer for 5 min. Protein concentration was determined by Bradford method. Equal amount (30 μg/lane) of protein samples were run on an SDS polyacrylamide gel. The proteins were transferred onto nitrocellulose membrane and blocked with 5% milk in PBS-0.05%Tween. The membrane was incubated with the primary antibody for cleaved caspase-3 (ISIS, Cat# 9664) or β-Actin (Sigma, Cat# A-5060) for 2 h at room temperature or overnight at 4°C. The membrane was washed 3 times with PBS-0.05% Tween and further incubated in appropriate horseradish peroxidase-conjugated secondary antibody (Pierce) for 90 min. Signals were detected using Western Lightening Plus ECL (Perkin Elmer).

### Statistical analysis

The data are expressed as mean ± S.D. for three independent experiments. Differences between the treatment groups were assessed using two-tailed unpaired student’s t-tests. The values with a *p < 0.05* were considered statistically significant.
